# Mitochondrial Reactive Oxygen Species and Lytic Programmed Cell Death in Acute Inflammation

**DOI:** 10.1089/ars.2022.0209

**Published:** 2023-10-16

**Authors:** Sergio Rius-Pérez, Salvador Pérez, Michel B. Toledano, Juan Sastre

**Affiliations:** ^1^ Department of Physiology, Faculty of Pharmacy, University of Valencia, Spain.; ^2^ Institute for Research in Biomedicine (IRB Barcelona), The Barcelona Institute of Science and Technology, Barcelona, Spain.; ^3^ RLT-Media Consulting, Boulogne-Billancourt, France.

**Keywords:** mitochondria, hydrogen peroxide, superoxide anion, necroptosis, ferroptosis, pyroptosis

## Abstract

**Significance:**

Redox signaling through mitochondrial reactive oxygen species (mtROS) has a key role in several mechanisms of regulated cell death (RCD), necroptosis, ferroptosis, pyroptosis, and apoptosis, thereby decisively contributing to inflammatory disorders. The role of mtROS in apoptosis has been extensively addressed, but their involvement in necrotic-like RCD has just started being elucidated, providing novel insights into the pathophysiology of acute inflammation.

**Recent Advances:**

p53 together with mtROS drive necroptosis in acute inflammation through downregulation of sulfiredoxin and peroxiredoxin 3. Mitochondrial hydroorotate dehydrogenase is a key redox system in the regulation of ferroptosis. In addition, a noncanonical pathway, which generates mtROS through the Ragulator-Rag complex and acts *via* mTORC1 to promote gasdermin D oligomerization, triggers pyroptosis.

**Critical Issues:**

mtROS trigger positive feedback loops leading to lytic RCD in conjunction with the necrosome, the inflammasome, glutathione depletion, and glutathione peroxidase 4 deficiency.

**Future Directions:**

The precise mechanism of membrane rupture in ferroptosis and the contribution of mtROS to ferroptosis in inflammatory disorders are still unclear, which will need further research. Mitochondrial antioxidants may provide promising therapeutic approaches toward acute inflammatory disorders. However, establishing doses and windows of action will be required to optimize their therapeutic potential, and to avoid potential adverse side effects linked to the blockade of beneficial mtROS adaptive signaling. *Antioxid. Redox Signal.* 39, 708–727.

**Tribute**

**Figure:**
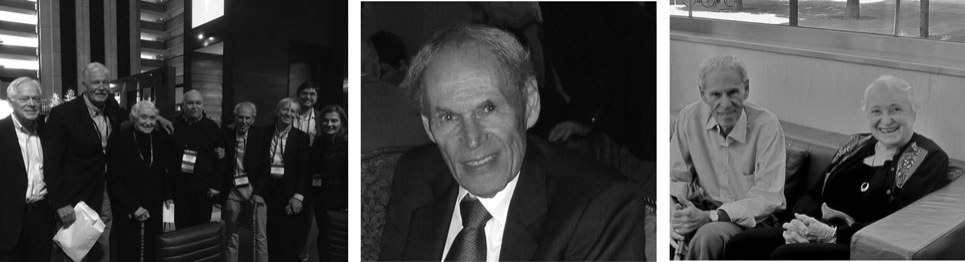
Professor Lester Packer was, indeed, one of the great pioneers in the field of Free Radicals and Antioxidants. He founded the Oxygen Club of California (OCC), served as President for many years, and was President of Society for Free Radical Research International (SFRR-I). He also organized numerous international meetings all over the world always exhibiting exceptional interpersonal skills, and greatly promoting the scientific career of many young investigators. I had the great opportunity to personally enjoy his enthusiasm for excellence in science in many OCC meetings, and particularly I had the honor of organizing together with Josiane Cillard, João Laranjinha, and Enrique Cadenas the OCC World Congress in Valencia in 2015, in his last visit to Europe. Professor Lester Packer will forever remain a reference for all of us, and we are indebted to him for pursuing the investigation in this exciting area of redox signaling and antioxidants.

## Regulatory Cell Death

In 1965, Lockshin and Williams observed cells dying during the metamorphosis of the silkworm *via* a process named “programmed cell death” (Lockshin and Williams, 1965). These authors used the term “programmed” because cells were programmed to die according to the “construction manual” for insect development (Nagata and Tanaka, 2017). In 1972, a similar cell death process was observed in human tissues (Kerr et al., 1972) and the term “apoptosis” was then coined to describe a specific morphological aspect of cell death characterized by the rounding-up of the cell, retraction of pseudopods, reduction of cellular volume (pyknosis), chromatin condensation, nuclear fragmentation (karyorrhexis), without or only very subtle ultrastructural modifications of cytoplasmic organelles, and maintenance of plasma membrane integrity until the final stages of the process culminating in engulfment of the apoptotic cell by phagocytes (Kroemer et al., 2009).

This type of cell death contrasted with “necrotic cell death,” morphologically characterized by a gain in cell volume (oncosis), swelling of organelles, plasma membrane rupture, and subsequent loss of intracellular contents (Kroemer et al., 2009).

Until the beginning of the 21st century, necrosis was believed to be merely an accidental and uncontrolled form of cell death. However, accumulating evidence proved that many cases of what was considered necrosis were, in fact, a tightly regulated form of cell death (Kroemer et al., 2009; Wang et al., 2018). Consequently, the cell death classification system had to discriminate between programmed forms of necrosis and the accidental form of necrosis (Galluzzi et al., 2015; Galluzzi et al., 2012; Kroemer et al., 2009).

Today, the Nomenclature Committee on Cell Death distinguishes two mutually exclusive types of cell death: accidental cell death (ACD) and regulated cell death (RCD) (Galluzzi et al., 2015). ACD is a biologically uncontrolled process, whereas RCD involves signaling cascades and effector mechanisms (Tang et al., 2019). While RCD can be pharmacologically and/or genetically modulated, ACD, generally caused by severe insults, including physical, chemical, and mechanical aggressions, cannot be prevented (Galluzzi et al., 2015).

**FIG. 1.**
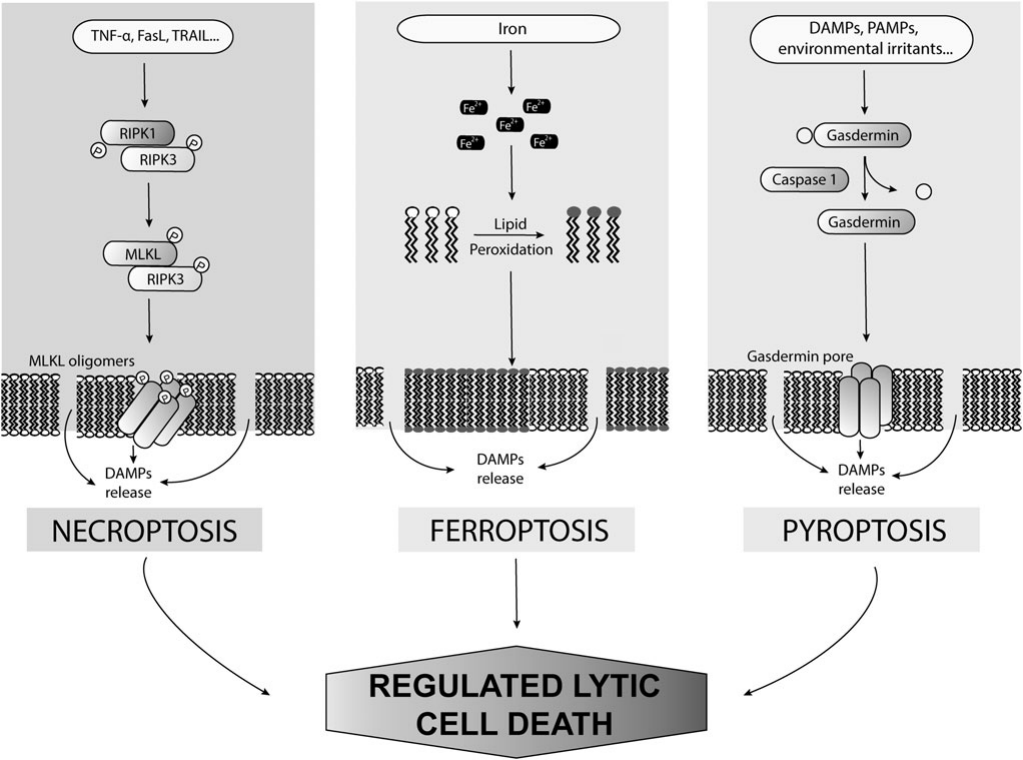
**Types of regulated lytic cell death**. DAMPs, damage-associated molecular patterns; FasL, Fas ligand; MLKL, pseudokinase mixed-lineage kinase domain–like; PAMPs, pathogen-associated molecular patterns; RIPK1/3, receptor-interacting protein kinases 1 and 3; TNFα, tumor necrosis factor α; TRAIL, TNF-related apoptosis-inducing ligand.

During the past decades, many novel forms of nonapoptotic RCD have been identified, including necroptosis, pyroptosis, ferroptosis, entotic cell death, parthanatos, lysosome-dependent cell death, autophagy-dependent cell death, alkaliptosis, and oxeiptosis (Tang et al., 2019). We focus here on lytic RCD in acute inflammation, with a special emphasis on necroptosis, ferroptosis, and pyroptosis (Fig. 1). These three cell death types are highly relevant to the pathophysiology of multiple diseases, and, in particular, inflammatory disorders (Chen et al., 2021b; Choi et al., 2019).

Necroptosis and pyroptosis are clearly relevant to the cell death occurring during viral infections, and their mechanisms have been elucidated (Oberst, 2016; Tang et al., 2019), in contrast to ferroptosis the physiological significance of which is still unclear as are the signaling events that trigger its induction (Jiang et al., 2021).

Cellular redox mechanisms and control pathways critically influence cell survival-death decisions (Ueda et al., 2002). Although oxidative stress is considered as a decisive factor in the trigger of cell death (Ryter et al., 2007), various redox signals also contribute to fine-tuning cell survival or death decisions (Benhar, 2020; Trachootham et al., 2008).

As one of the main sites of reactive oxygen species (ROS) production, mitochondria is relevant to the mechanisms that trigger cell death programs (Collins et al., 2012). A large variety of intra- and extracellular cues that can signal cell fate decisions promote or prevent cell death by modulating mitochondrial function and structure (Sedlackova and Korolchuk, 2019). Indeed, most of the biochemical events associated with cell death, including irreversible metabolic changes and activation of cell death effectors, eventually trigger mitochondrial outer membrane permeabilization, a decisive step that delimits the frontier between cell survival and death (Kroemer et al., 2007).

We address here the role of mitochondrial ROS (mtROS) in the regulation of lytic programmed cell death in the context of acute inflammation. Literature over the past 15 years was identified in PubMed database by searching the following keywords: necroptosis, ferroptosis, pyroptosis, mitochondrial ROS, mitochondrial antioxidants, and inflammation. Relevant references to the topic cited by publications retrieved using this search strategy were also consulted.

## Necroptosis

Necroptosis, a programmed form of cell death morphologically similar to necrosis, is triggered by the engagement of cell surface death receptors, such as the Fas cell surface death receptor (Fas), tumor necrosis factor alpha receptor 1 (TNFR1), interferon receptors (IFNRs), Toll-like receptors, and intracellular RNA- or DNA-sensing molecules (Choi et al., 2019). The receptor-interacting protein kinases 1 and 3 (RIPK1 and RIPK3) and the pseudokinase mixed-lineage kinase domain–like (MLKL) are the key proteins that execute necroptosis, which may occur when apoptosis is impaired (Vanden Berghe et al., 2014).

**FIG. 2.**
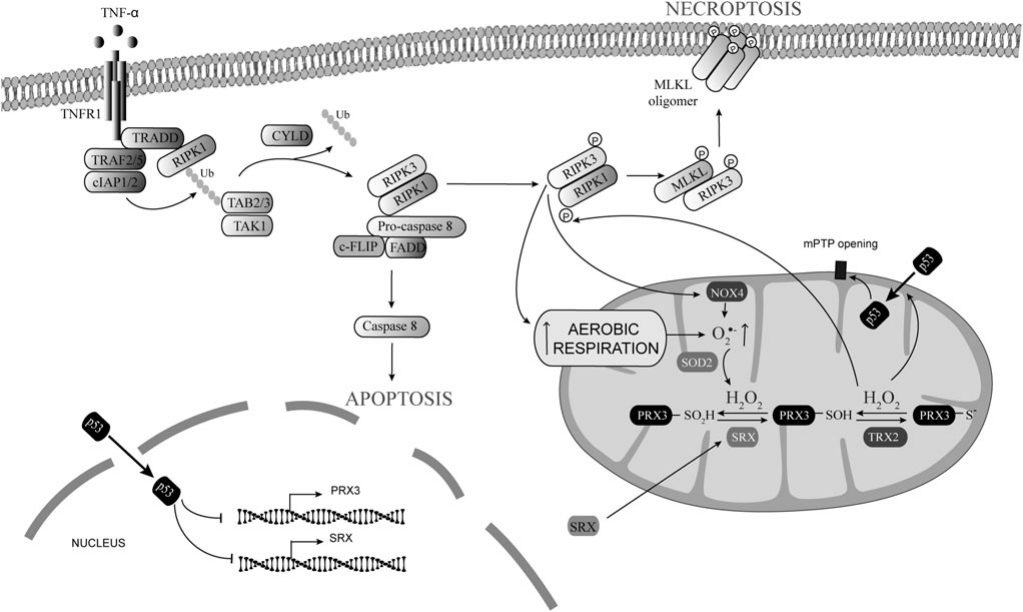
**Regulation of necroptosis by mtROS.** Increased aerobic respiration, mitochondrial translocation of NOX4, and p53-dependent SRX and PRX3 downregulation increased mtROS. Increased mtROS cause mitochondrial translocation of p53 and RIP1K phosphorylation. Phosphorylated RIPK1 recruits and phosphorylates RIPK3 into the necrosome. Phosphorylated RIPK3 then phosphorylates MLKL, triggering the formation of MLKL oligomers that translocate and destabilize the plasma membrane. MLKL, pseudokinase mixed-lineage kinase domain–like; mtROS, mitochondrial reactive oxygen species; NOX, NADPH oxidases; PRX3, peroxiredoxin 3; RIPK1/3, receptor-interacting protein kinases 1 and 3; SRX, sulfiredoxin.

The tumor necrosis factor α (TNFα)-dependent signaling pathway exemplifies the molecular switches that determine survival *versus* cell death by apoptosis or necroptosis (Fig. 2). On TNFR1 stimulation, RIPK1, TNFR1-associated death domain (TRADD), tumor necrosis factor receptor–associated factor (TRAF)2/TRAF5, and cellular inhibitor of apoptosis protein (cIAP)1/cIAP2 interact to form a membrane-signaling complex known as complex I (Vanden Berghe et al., 2014).

Within complex I, cIAPs-dependent ubiquitination of RIPK1 causes recruitment of the inhibitor of I-κB kinase complex, leading to activation and nuclear translocation of nuclear factor kappa-light-chain-enhancer of activated B cells (NF-κB) (Choi et al., 2019). NF-κB-dependent cell survival response is critical to counteract activation of the cytosolic pro-apoptotic complex IIa formed by TRADD-FAS-associated death domain protein (FADD) and caspase-8 (Dondelinger et al., 2016).

However, when activation of NF-κB by complex I is downregulated, TRADD dissociates from complex I and engages FADD and pro-caspase-8 in the cytosol, causing the formation of complex IIa (Micheau and Tschopp, 2003). The formation of complex IIa activates caspase-8, which triggers activation of the apoptosis effectors caspase-3 and caspase-7 (Dondelinger et al., 2016).

Alternatively, when ubiquitination of RIPK1 is disrupted in complex I as a consequence of cIAPs inhibition or CYLD upregulation, a protein that removes polyubiquitin from RIPK1, RIPK1 dissociates from complex I and interacts with FADD and pro-caspase-8 (Dondelinger et al., 2016; Kist et al., 2021). The formation of this complex also promotes activation of apoptosis in a caspase-8-dependent manner and thus, it is considered a pro-apoptotic RIPK1 kinase-dependent version of complex II, which is usually denoted as complex IIb (Wegner et al., 2017).

In complex IIb, activated caspase-8 cleaves and inactivates RIPK1 as well as RIPK3, preventing necroptosis activation (Newton et al., 2019; Oberst et al., 2011). When caspase-8 is inhibited, the cleavage of RIPK1 and RIPK3 is repressed and hence, RIPK1 and RIPK3 can phosphorylate each other stabilizing a pro-necroptotic complex called the necrosome (He et al., 2009; Weinlich et al., 2017).

Phosphorylated RIPK3 then phosphorylates MLKL, which triggers the formation of MLKL oligomers that localize to and destabilize the plasma membrane (Cai et al., 2014; Dondelinger et al., 2014; Newton and Manning, 2016; Wang et al., 2014a). Phosphatidylinositol transfer protein alpha (PITPα), which transfers phosphatidylinositol between membranes, and HSP90 interact with MLKL, facilitating its oligomerization and plasma membrane localization (Jacobsen et al., 2016; Jing et al., 2018). The precise mechanisms that regulate MLKL membrane localization and subsequent disruption of plasma membrane integrity are still unclear (Li et al., 2021). According to the model of TNFα-mediated necroptosis in HEK293T and L929sAhFas cell lines, MLKL binds phosphatidylinositol phosphate through positive charges present in the four-helical bundle domain of its N-terminal region, thereby disrupting plasma membrane directly (Dondelinger et al., 2014).

Nevertheless, the studies on TNFα-induced necroptosis in HT29, FADD^−/−^ Jurkat, J774A.1, U937, and L929 cell lines suggest that MLKL recruits and primes Ca^2+^ and Na^+^ ion channels, thereby causing osmotic pressure increase and membrane rupture (Cai et al., 2014; Chen et al., 2014b). Although several studies have pointed that the latter model cannot be correct, since the aforementioned ion channels are not essential for necroptosis, the effect of MLKL on ion influx might be relevant (Flores-Romero et al., 2020; Ousingsawat et al., 2017; Xia et al., 2016).

Alternatively, MLKL oligomers themselves may instead act as a channel (Xia et al., 2016). It, thus, remains to be elucidated whether membrane permeability is the result of MLKL insertion into the lipid bilayer or alternatively MLKL formation of pores that act as ion channels, or possibly to both mechanisms (Flores-Romero et al., 2020).

Importantly, MLKL trafficking and accumulation at the plasma membrane control the kinetics and threshold for necroptosis (Samson et al., 2020). Activated MLKL generates bubbles with exposed phosphatidylserine that are released from the surface (Li et al., 2021). The endosomal sorting complex required for transport (ESCRT)-III machinery is required for the formation of these bubbles and controls the duration of plasma membrane integrity, allowing necroptotic cells to generate and release signaling molecules before membrane rupture (Gong et al., 2017).

In fact, ESCRT-III-mediated bubble formation can sustain cell survival when MLKL activation is reduced or reversed (Gong et al., 2017). Consequently, activation of MLKL is not a “point-of-no-return” for cell survival since cells harboring active MLKL can be resuscitated by maintaining ESCRT activity (Gong et al., 2017). Accordingly, membrane bubbling appears as a transient step during necroptosis since it occurs during the induction phase of necroptosis but decreases just before cell death (Gong et al., 2017; Samson et al., 2020).

Activated MLKL accumulates at the plasma membrane until a membranolytic threshold is reached, which causes membrane blowout and cell death (Samson et al., 2020). At this stage, MLKL traffics together with tight junction proteins to the cell periphery *via* Golgi-microtubule-actin-dependent mechanisms, thus accumulating at sites of intercellular junctions. The accumulation of phosphorylated MLKL at intercellular junctions accelerates necroptosis of neighboring cells, thereby propagating necroptotic signaling within the tissue (Samson et al., 2020).

### Necroptosis and inflammation

Necroptosis exhibits typical morphological features of necrosis, including organelle swelling, plasma membrane rupture, cell lysis, and leakage of intracellular components, which may propagate secondary inflammatory responses (Choi et al., 2019). When released during cell death, intracellular contents can act as damage-associated molecular patterns (DAMPs) that induce an inflammatory response by interacting with and activating different innate immune effectors (Kaczmarek et al., 2013).

These DAMPs include high mobility group box 1 (HMGB1)—a nonhistone chromatin-binding protein involved in transcriptional regulation (Janko et al., 2014), S100 proteins, interleukin 33 (IL-33), mitochondrial DNA (mtDNA), and cell-free DNA (cfDNA) (Afonso et al., 2015; Chen et al., 2021a; Duprez et al., 2011; Kovalenko et al., 2009; Liu et al., 2018b; Negroni et al., 2017; Rius-Pérez et al., 2022; Wen et al., 2020).

Hence, necroptosis must have a decisive role in inflammatory diseases such as sepsis, stroke, cardiac ischemia/reperfusion injury, inflammatory bowel disease, acute kidney injury, acute pancreatitis, nonalcoholic steatohepatitis, chronic obstructive pulmonary disease, and skin inflammation (Choi et al., 2019; Khoury et al., 2020; Newton and Manning, 2016; Weinlich et al., 2017). The specific pathophysiological role of each DAMP in modulating immune responses associated with necroptosis remains to be clarified though (Choi et al., 2019).

DAMPs can be passively released by membrane rupture due to necroptosis, but they can also be actively secreted by living cells under life-threatening stress (Murao et al., 2021; Vénéreau et al., 2015). The mechanisms that govern DAMPs release in necroptotic cells are not fully understood. Using a fluorescence resonance energy transfer biosensor, termed SMART, in combination with imaging of the release of nuclear DAMPs and live-cell imaging for secretion activity, two different modes of HMGB1 release have been revealed: “a burst-mode” and “a sustained-mode” (Murai et al., 2018).

Charged multivesicular body protein 4B (CHMP4B)—a key component of the ESCRT-III complex that preserves plasma membrane integrity during necroptosis execution (Gong et al., 2017)—contributes toward maintaining the sustained-mode of HMGB1 release, possibly by actively repairing plasma membrane (Murai et al., 2018). In addition, this experimental approach showed that HMGB1 was rapidly released from necroptotic cells through two sequential steps: the first step is nucleo-cytoplasmic translocation, and the second step is the extracellular release from the cytoplasm (Murai et al., 2018). This observation suggests that the nuclear membrane is damaged before the cytoplasmic membrane becomes ruptured.

There is no evidence that necroptosis is accompanied by release of a specific set of DAMPs. In fact, many DAMPs released from cells undergoing necroptosis coincide with those released during other types of cell death such as apoptosis, pyroptosis, ferroptosis, and NETosis (Murao et al., 2021). Nevertheless, several studies suggest that the compositions and signatures of DAMPs might be qualitatively and quantitatively different as a function of the different biochemical pathway that control each type of cell death (Choi et al., 2019; Tanzer et al., 2020).

For instance, the full-length biologically active IL-33 released during necroptosis is different from the nonimmunological form of IL-33 released during apoptosis produced by caspase-dependent proteolysis of IL-33, since caspase activity is abrogated during necroptosis execution (Lüthi et al., 2009; Shlomovitz et al., 2019). Furthermore, H3 is not or only marginally released by necroptotic cells (Murai et al., 2018), whereas histones, which are highly associated with caspase-dependent DNA fragmentation, are released from apoptotic cells (Chen et al., 2014a). Release of the calreticulin—an “eat me” signal for dendritic cells—is also dependent on the activity of caspase-8 and therefore, this DAMP is associated with apoptosis but not with necroptosis (Panaretakis et al., 2009).

Of note, macrophages use different internalization mechanisms, hence exhibiting different kinetics to clear out apoptotic and necrotic/necroptotic cells (Brouckaert et al., 2004; Krysko et al., 2006). Apoptotic bodies are cleared out through the formation of tight phagosomes, whereas necrotic/necroptotic cells are internalized by a macropinocytic mechanism, which involves the formation of multiple ruffles directed toward necrotic debris (Krysko et al., 2006).

Hence, phagocytosis of apoptotic cells is quantitatively and kinetically more efficient than phagocytosis of necrotic/necroptotic cells, which suggest that necroptotic cells could release more DAMPs than apoptotic cells, therefore promoting a stronger inflammatory response (Brouckaert et al., 2004).

Under basal conditions, HMGB1 is not tightly bound to chromatin, and therefore it can passively diffuse from the nucleus to the extracellular space in necrotic cells (Janko et al., 2014). However, during apoptosis, caspase-dependent acetylation of histones and chromatin condensation cause attachment of HMGB1 to chromatin, which limits HMGB1 release from apoptotic cells (Kaczmarek et al., 2013; Scaffidi et al., 2002; Taylor et al., 2008).

The ROS can modify redox-sensitive DAMPs and, consequently, their immunogenicity (Koenig and Buskiewicz-Koenig, 2022). By modification of the three conserved redox-active cysteine (Cys^23^, Cys^45^, and Cys^106^) that it carries, HMGB1 is found in three different oxidation states that are designated as “reduced HMGB1” (with all three cysteine residues in the thiol state), “disulfide HMGB1” (with an intramolecular disulfide bond between Cys^23^ and Cys^45^), and “sulfonic HMGB1” (with all three cysteines in the hyperoxidized sulfonic acid state).

**FIG. 3.**
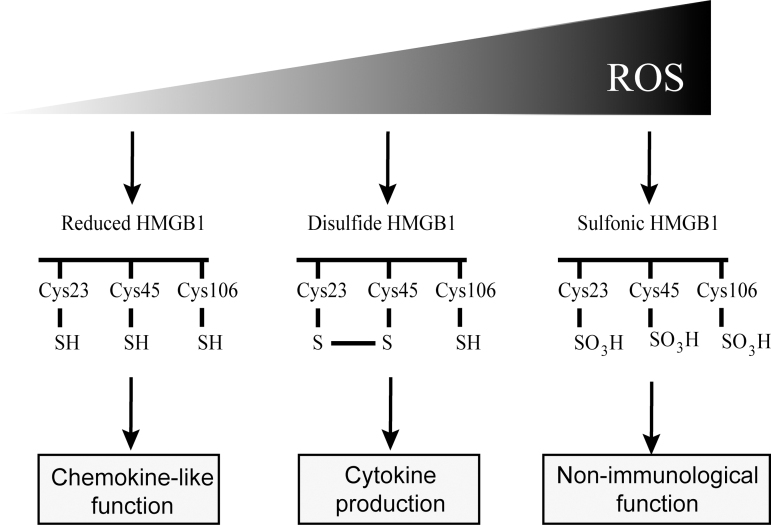
**Different redox states of HMGB1.** HMGB1 is found in three different oxidation states: reduced HMGB1 (with Cys23, Cys45, and Cys106 residues in the thiol state), “disulfide HMGB1” (with an intramolecular disulfide bond between Cys23 and Cys45), and “sulfonic HMGB1” (with all three cysteines in the hyperoxidized sulfonic acid state). Reduced HMGB1 exhibits chemokine-like function, disulfide HMGB1 promotes cytokine production, whereas in contrast, sulfonic HMGB1 does not exert any immunological function. HMGB1, high mobility group box 1.

These redox HMGB1 conformers have different immunogenic properties (Fig. 3). Reduced HMGB1 exhibits chemokine-like function by forming a complex with CXCL12 promoting immune cell migration, disulfide HMGB1 activates NF-κB and promotes cytokine production in macrophages (Venereau et al., 2012), whereas in contrast, sulfonic HMGB1 does not exert any immunological function (Kazama et al., 2008).

HMGB1 released from necroptotic cells was shown to exert an effect in mouse inflammation models of chronic obstructive pulmonary disease, acute kidney injury, spinal cord injury, psoriatic inflammation, and inflammatory myopathies (Chen et al., 2021a; Duan et al., 2020; Fan et al., 2016; Kamiya et al., 2022; Liu et al., 2018b). However, HMGB1 was not detected in plasma of mouse models of necroptosis in acute pancreatitis and neonatal *Ripk1^−^*^/*−*^ lethal systemic inflammation (Rickard et al., 2014; Rius-Pérez et al., 2022).

Another necroptotic DAMP, cfDNA, was, however, detected in the plasma of mice with acute pancreatitis in parallel with the presence of necroptosis in the pancreas, indicating that cfDNA levels but not HMGB1 levels may serve as a marker of necroptosis (Rius-Pérez et al., 2022). In *Ripk1^−/−^* mice, IL-1α and IL-33 levels increased in plasma and correlated better than HMGB1 with the extensive necroptosis found in these mice (Rickard et al., 2014). Nevertheless, the kinetics of HMGB1 release may differ between different cell types (Murai et al., 2018), so a detailed time-course of plasma HMGB1 levels would be required to establish its exact relevance in these inflammatory models.

Although necroptosis is highly pro-inflammatory, it might also paradoxically cause anti-inflammatory effects in specific pathophysiological contexts in which elimination of necroptotic cells could be beneficial (Kearney and Martin, 2017). In particular, necroptosis can serve to eliminate pathogen-infected cells during infectious diseases (Choi et al., 2019).

This idea is corroborated by the high *Staphylococcus aureus* load, impaired ability to limit IL-1β production, and excessive inflammation of *Mlkl^−^*^/*−*^ mice infected by this pathogen, and by the increased bacterial load of mice treated with RIPK1 or RIPK3 inhibitors in a model of sepsis (Kitur et al., 2016). These findings indicate that necroptosis contributes to innate immunity during infection, and that its inflammatory effects are complex and highly dependent on the net balance between the synthesis of pro-inflammatory cytokines and the level of DAMPs released by dying cells.

The ROS generation triggered by RIPK3 is necessary for TNFα-induced necroptosis (Zhang et al., 2009a). RIPK3 activates the enzymes glycogen phosphorylase, glutamate-ammonia ligase, and glutamate dehydrogenase 1, and, in turn, these key enzymes enhance TNFα-induced ROS production (Zhang et al., 2009b). Indeed, increasing glucose utilization by breaking down glycogen or the use of glutamine and glutamate as energy substrates fuels the mitochondrial respiratory chain generating mtROS at the ubisemiquinone site in cells incubated with cytotoxic doses of TNFα (Zhang et al., 2009b).

NADPH oxidases (NOX) are also involved in the production of ROS during necroptosis. By forming a complex with TRADD, RIPK1, and RAC1, the nonmitochondrial NOX1 produces superoxide in necroptotic L929 and MEF cells incubated with TNFα (Kim et al., 2007). However, TNFα-induced necroptosis was only partially reduced in L929 cells on NOX1 inhibition (Kim et al., 2007). Nevertheless, treatment with the ROS scavengers butylated hydroxyanisole (BHA), *N*-acetylcysteine, TEMPOL, or MitoQ efficiently dampened necroptosis (Kim et al., 2007; Yang et al., 2018). Therefore, these results suggest that other sources of superoxide—presumably mitochondrial sources—are also involved in ROS generation during necroptosis.

### Regulation of necroptosis by mtROS

In addition to the prototypical TNFα-dependent pathway for necroptosis activation, novel mechanisms have emerged that involve increased mtROS generation (Fig. 2) (Rius-Pérez et al., 2022; Roca et al., 2022). mtROS activate RIPK1 autophosphorylation on Ser161 *via* oxidation of three specific cysteines (Cys257, Cys268, and Cys586), which promotes RIPK3 recruitment to the necrosome (Zhang et al., 2017).

Furthermore, ROS-dependent disulfide bond formation between MLKL subunits is necessary to induce necroptosis (Liu et al., 2017). In fact, thioredoxin-1 actively maintains MLKL in a reduced inactive state suppressing disulfide bond-dependent MLKL polymer formation and necroptosis (Reynoso et al., 2017).

RIPK3-induced mtROS generation appears associated with aerobic respiration during necroptosis, as suggested by the following observations. ROS production during TNFα-induced necroptosis was prevented by treatment with BHA or MitoQ (Yang et al., 2018). Activated RIPK3 in the necrosome phosphorylates the PDC-E3 subunit of the mitochondrial matrix pyruvate dehydrogenase complex (PDC) to increase aerobic respiration and ROS generation, which then induces necroptosis (Yang et al., 2018). RIPK3 must localize to mitochondria to phosphorylate PDC-E3 (Joplin et al., 1995).

Interestingly, MLKL is required for PDC activation, and MLKL phosphorylation is also required for mitochondrial localization of the necrosome (Yang et al., 2018). It has, thus, been proposed that the high affinity binding of MLKL to cardiolipin—a lipid component of the mitochondrial inner membrane (Paradies et al., 2014)—would be the driving force bringing RIPK3 to the inner side of mitochondria to regulate PDC and aerobic respiration (Wang et al., 2014a; Yang et al., 2018).

Remarkably, in mycobacterium-infected macrophages, excess TNFα increased cellular glutamine uptake (Roca et al., 2022). Excess glutamine enhanced succinate production by the Krebs cycle, which triggered a large increase in mtROS generation by reverse electron transport (RET) through complex I leading to necroptosis (Roca et al., 2022).

Interestingly, RIPK3 knockdown inhibited TNFα-induced succinate production, mtROS generation, and subsequent necroptosis, supporting the idea of the RIPK3-dependent regulation of aerobic respiration (Roca et al., 2022; Yang et al., 2018; Zhang et al., 2009b). Intriguingly, knockdown of mitochondrial phosphatase phosphoglycerate mutase 5 (PGAM5), one of RIPK3 substrates, produced effects similar to those of the RIPK3 knockdown in preventing necroptosis in mycobacterium-infected macrophages (Roca et al., 2022).

Furthermore, upon induction of necroptosis, PGAM5 recruited and activated the mitochondrial fission factor dynamin-related protein 1 (DRP1), which caused mitochondrial fragmentation (Wang et al., 2012), and necroptosis inhibition by necrostatin-1 decreased DRP1 levels (Zhang et al., 2013). Phosphorylated DRP1 localizes to the mitochondrial outer membrane, where it engages the mitochondrial fission protein FIS1, inducing mitochondrial fission, and ROS accumulation (Wang et al., 2014b).

In vesicular stomatitis virus-infected macrophages, RIPK1 phosphorylates DRP1 at Ser616, and binds DRP1 in the presence of RIPK3, thus forming a ternary complex (Rayamajhi and Miao, 2014; Wang et al., 2014b). Importantly, the anti-diabetic drug metformin abrogated necroptosis by blocking RET-generated mtROS at complex I and/or by acting on mitochondrial dynamics through inhibition of DRP1 (Roca et al., 2022; Zhang et al., 2019b).

Considering that mitochondrial fission has been associated with reduced electron transport efficiency, increased aerobic glycolysis, and augmented ROS generation (Buck et al., 2016; Yu et al., 2006), further investigation about how PGAM5 and mitochondrial fission regulate necroptosis is warranted.

Remarkably, when the inflammasome is activated in leukine-rich repeat kinase 2 (*Lrrk2*)^G2019S^ macrophages—a gain-of-function allele associated with a more severe inflammatory response against *Mycobacterium tuberculosis*—disruption of mitochondrial membranes by the pore-forming protein gasdermin D elevates mtROS to trigger necroptosis instead of pyroptosis (Weindel et al., 2022). Hence, mtROS may act as a switch between necroptosis and pyroptosis.

On the other hand, hyperglycemia caused a shift from apoptosis to necroptosis *via* mtROS-dependent oxidation of RIPK1, which exacerbated neonatal hypoxia-ischemia brain injury in mice (Deragon et al., 2020). These results highlight the critical role of mtROS to modulate cell death fate according to the cellular redox status.

In the widely used mouse fibrosarcoma L929 cells model of necroptosis induced by TNFα, necroptosis was accompanied by modest intracellular ROS production (Vanlangenakker et al., 2011). In the absence of the inhibitor of apoptosis proteins-1/-2 (cIAP1 and cIAP2), TNF-induced necroptosis occurred by RIPK1/3-mediated ROS production (Vanlangenakker et al., 2011).

Remarkably, repression of the NADH dehydrogenase (ubiquinone) 1 beta subcomplex 8 (NDUFB8), a subunit of mitochondrial complex I, markedly diminished necroptotic cell death induced by TNFα or by TNFα plus the IAP antagonist BV6 (Vanlangenakker et al., 2011). Hence, complex I activity seems critical to regulate mtROS generation during necroptosis. Further, NDUFB8 appeared nitrated in endothelial cells undergoing necroptosis, and this nitration was prevented by overexpression of mitochondrial superoxide dismutase (Davis et al., 2010).

In kidney tubular epithelial cells, RIPK3 promotes oxidative stress together with inhibition of mitochondrial complexes I and III, and upregulation of NOX4 (Sureshbabu et al., 2018). Interestingly, RIPK3 and NOX4 form a complex in response to pro-inflammatory stimuli, which promotes the accumulation of NOX4 into the mitochondria (Sureshbabu et al., 2018).

RIPK3-dependent signaling also triggers mitochondrial depolarization, reduced expression of mitochondrial complex subunits, and increased ROS production during kidney inflammation (Sureshbabu et al., 2018). Therefore, mitochondrial NOX4 and the downregulation of mitochondrial respiratory complexes by RIPK3 contribute to ROS generation during necroptosis in the inflamed kidney.

As recently established, the mitochondrial peroxiredoxin (PRX), PRX3, and the PRX sulfinic reductase, sulfiredoxin (SRX), have a critical role in the regulation of necroptosis through mtROS (Rius-Pérez et al., 2022). At the early stage of acute pancreatitis, SRX became upregulated and accumulated into mitochondria from its cytoplasmic location, thus preventing PRX3 hyperoxidation.

In the late stage of pancreatitis, at the time of necroptosis activation, SRX was then dramatically downregulated, PRX3 became hyperoxidized, and H_2_O_2_ levels were increased (Rius-Pérez et al., 2022). During pancreatitis in mice lacking SRX, necroptosis occurred much faster, and could be prevented by the mitochondrial antioxidant MitoTEMPO, therefore indicating the critical importance of mtROS in necroptosis activation, and hence the role of PRX3 and SRX in mitigating mtROS levels (Rius-Pérez et al., 2022).

Considering that PRX3 can also act as ONOO^−^ oxidoreductase, exhibiting kinetic constants toward this oxidant in the range of those measured for H_2_O_2_ (Cox et al., 2009; De Armas et al., 2019), this enzyme must also serve to combat the mitochondrial nitrosative stress seen in necroptotic cells (Davis et al., 2010).

SRX protein levels during necroptosis appear to be controlled by p53, which accumulates in mitochondria. In the pancreas of mice with acute pancreatitis lacking p53, SRX downregulation was lost, hyperoxidized PRX3 was quasi undetectable as a consequence of high SRX expression, and MLKL phosphorylation was not detected (Rius-Pérez et al., 2022). Reciprocally, mtROS stimulate the mitochondrial translocation of p53 required for the execution of necroptosis, as seen in the pancreas of mice with pancreatitis and also in obese mice (Rius-Pérez et al., 2022).

It has also been shown that when p53 accumulate in the mitochondrial matrix, it forms a complex with cyclophilin D (CypD), which may trigger the opening of mitochondrial permeability transition pores (mPTP) and necrosis (Guo et al., 2014; Vaseva et al., 2012). Similarly, when phosphorylated at serine 23, mitochondrial p53 binds CypD, thereby causing necrosis in mouse cortical neurons (Pei et al., 2014).

We found that during pancreatitis, mice lacking SRX had a marked accumulation of p53 into mitochondria, in addition to displaying elevated levels of mitochondrial H_2_O_2_ and accelerated necroptosis (Rius-Pérez et al., 2022). Treatment with MitoTEMPO decreased mitochondrial translocation of p53 and abrogated necroptosis in these mice, thus indicating the importance of mtROS in the mitochondrial translocation of p53 (Rius-Pérez et al., 2022).

The requirement of p53 for regulated necrosis has also been observed during normal spermatogenesis in *Drosophila melanogaster* (Napoletano et al., 2017), and in mammalian cardiomyocytes exposed to H_2_O_2_, a requirement that involved the transcriptional upregulation of the long noncoding RNA necrosis-related factor (Wang et al., 2016a).

In summary, the decision between survival and necroptotic cell death during pancreatitis appears to be a function of mtROS levels, which are at least in part governed by a reciprocal regulation between p53 and the SRX-PRX3 antioxidant system, with p53 promoting an mtROS buildup by causing the inactivation of PRX3 by hyperoxidation through the downregulation of SRX, and the SRX-PRX3 system restraining p53 mitochondrial accumulation by decreasing mtROS levels (Rius-Pérez et al., 2022).

It is worth noting that activation of the necrosome is paralleled by an increased flux of calcium in the cytoplasm (González-Juarbe et al., 2017; Nomura et al., 2014; Sun et al., 2017). The accumulation of [Ca^2+^]_m_ is generally associated with increased mtROS production (Görlach et al., 2015), thus suggesting a potential [Ca^2+^]_m_-mediated regulation of mtROS during necroptosis.

Accordingly, TNFα increases Ca^2+^ influx into the mitochondria, which leads to augmented mtROS generation (Dada and Sznajder, 2011). Interestingly, the nine amino acid-residue peptide fragment—an antimicrobial peptide derived from lactoferrin—caused necroptosis in HL60 cells by increasing endoplasmic reticulum (ER) stress, [Ca^2+^]_c_, and mtROS levels (Lv et al., 2019).

Remarkably, treatment with MitoQ rescued these cells from necroptosis, suggesting that mtROS are part of the ultimate step in ER-stress mediated necroptosis induction (Lv et al., 2019). Importantly, [Ca^2+^]_m_ modulates the activity of several rate-limiting enzymes of the Krebs cycle, including PDC (Denton, 2009). Therefore, it is plausible that elevated mitochondrial calcium levels act synergistically with mitochondrial metabolism to enhance mtROS and necroptosis.

In addition, elevated [Ca^2+^]_m_ can also disturb F_1_F_0_-ATP synthase activity and cause opening of mPTP, two conditions tightly associated with elevated mtROS production and necroptosis (Faizan and Ahmad, 2021). However, mitochondria lacking the mitochondrial Ca^2+^ uniporter exhibited enhanced mPTP calcium sensitivity (Parks et al., 2019). Hence, the regulation of mPTP opening by [Ca^2+^]_m_ in the generation of mtROS and cell death during necroptosis deserves further investigation.

In contrast to the findings mentioned so far, some authors have reported a lack of mitochondrial translocation of RIPK3, and the existence of RIPK1/RIPK3-independent mechanisms in the regulation of ROS production during necroptosis (Vanlangenakker et al., 2011). Other authors have questioned the key role of mitochondria in necroptosis, as this type of cell death may occur in mitochondrial-depleted cells.

These contradictory findings could be rationalized by the differences in the response to TNFα exhibited in different cell types (Marshall and Baines, 2014; Tait et al., 2013). Indeed, ROS are not induced by TNFα in human colon cancer HT-29 cells (Yang et al., 2018), and ROS are dispensable for necroptosis in certain cell lines, such as endothelial SVEC or fibroblasts 3T3-SA cells (He et al., 2009; Tait et al., 2013). Interestingly, in cells with RIPK3-dependent regulation of mtROS generation, hypoxia inhibits necroptosis without altering RIPK1, RIPK3, and MLKL levels (Yang et al., 2018).

It is noteworthy that hypoxia induces pyruvate dehydrogenase kinase 1 expression, which phosphorylates PDC and inhibits PDC activity attenuating ROS generation (Kim et al., 2006). Hence, the coupling of aerobic respiration and mtROS generation with RIPK3 signaling could be a key mechanism in certain cell types to control the threshold for necroptosis induction according to the local oxygen concentrations (Kim et al., 2006; Yang et al., 2018).

## Ferroptosis

**FIG. 4.**
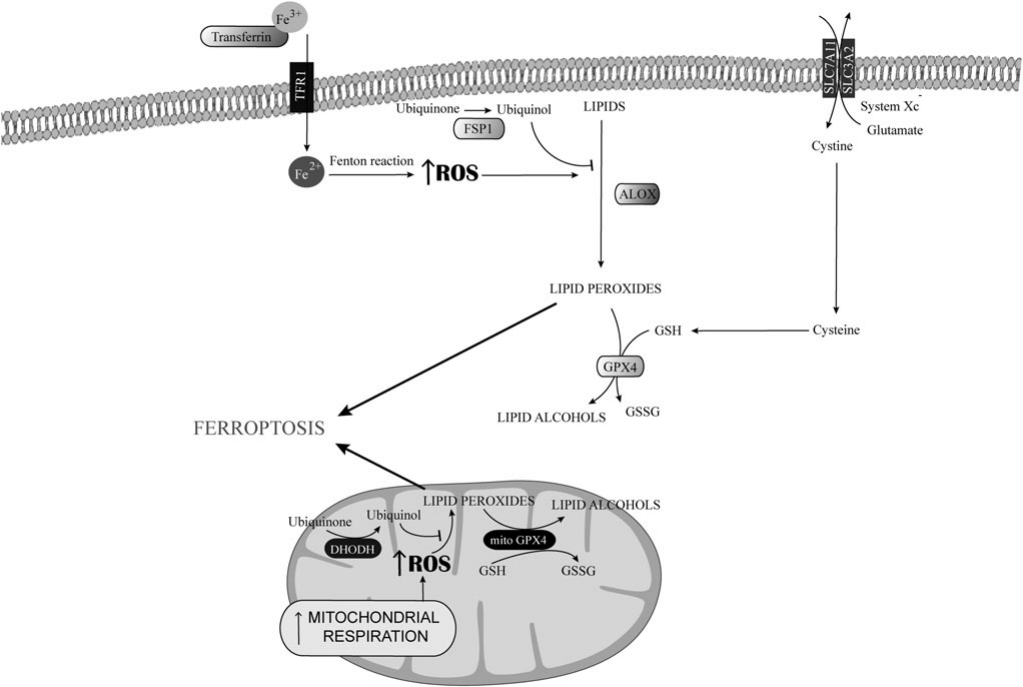
**Regulation of ferroptosis by mtROS.** Ferroptosis is an iron and ROS-dependent regulated form of cell death characterized by the formation of lipid hydroperoxides. The mtROS cause lipid peroxidation in the mitochondria, which contributes to ferroptosis. Cytosolic GPX4 and mitochondrial GPX4 prevent ferroptosis, directly converting lipid hydroperoxides to lipid alcohols in the plasma membrane and in the inner mitochondrial membrane, respectively. FSP1 and DHODH prevent ferroptosis by generating ubiquinol from ubiquinone to counteract oxidative lipid production into the plasma cell membrane and into the inner mitochondrial membrane, respectively. DHODH, hydroorotate dehydrogenase; FSP1, NAD(P)H/ferroptosis suppressor protein-1; GPX4, glutathione peroxidase 4.

Ferroptosis was discovered in 2012 by Stockwell as an iron-dependent lipid peroxidation process causing cell death, with morphological, biochemical, and mechanistic features markedly different from the other types of RCD (Dixon et al., 2012; Dixon and Stockwell, 2014; Galluzzi et al., 2018). Ferroptosis is characterized by lipid peroxidation and prevented by iron depletion (Fig. 4) (Dixon et al., 2012; Yang et al., 2016).

Iron depletion resulting from dysfunction of iron regulatory proteins (Dixon et al., 2012) prevented the induction of ferroptosis by arestin or other inducers, whereas autophagic degradation of ferritin promoted it by increasing cellular labile iron availability (Ye et al., 2020). Such an iron dependency is not fully understood. Excess labile iron availability by driving the nonenzymatic Fenton chain reaction presumably promotes ferroptosis by enhancing lipid peroxidation, but iron is also required in iron-dependent enzymes, such as lipoxygenase (LOX), an enzyme responsible for the lipid peroxidation that affects unsaturated fatty acids in the plasma membrane, which execute ferroptosis (Wenzel et al., 2017).

The glutathione-dependent enzyme glutathione peroxidase 4 (Gpx4) and system X_c_^−^ are essential in the control of ferroptosis (Zhang et al., 2021). System X_c_^−^ is a transmembrane protein complex that contains the subunits solute carrier family 7 member 11 (SLC7A11) and solute carrier family 3 member 2 (SLC3A2), which acts as a cystine/glutamate antiporter to provide the oxidized form of the amino acid cysteine (cystine) as a source of organic sulfur (Fig. 4) (Lewerenz et al., 2013).

Inhibition of system X_c_^−^ with erastin, sulfasalazine, or sorafenib triggers ferroptosis due to blockade of cystine uptake (Dixon et al., 2014; Jeong et al., 2013; Yu et al., 2019). However, whether cysteine deficiency causes ferroptosis by decreasing glutathione synthesis is the subject of discussion.

GPX4 is a selenoenzyme initially discovered by Ursini et al. (1982) on the basis of its ability to scavenge phospholipids hydroperoxides by reduction to their respective alcohols. GPX4 is the major enzyme catalyzing this reduction in and out of cell membranes (Yang et al., 2014). GPx4 knockout mice are highly susceptible to ferroptosis in different experimental models (Xie et al., 2016).

Pharmacological inhibition of GPx4 by RAS-selective lethal 3 (RSL3) prevented the induction of ferroptosis by arestin or other inducers, and other such compounds cause ferroptosis with parallel accumulation of lipid hydroperoxides (Yang et al., 2014). These studies highlight the importance of the X_c_^−^/GPx4 axis in the regulation of ferroptosis (Li et al., 2022).

In parallel with GPX4, the enzyme NAD(P)H/ferroptosis suppressor protein-1 prevents oxidative lipid production by providing electrons at the plasma cell membrane through the generation of ubiquinol (CoQH10) from ubiquinone (CoQ10), and hence protects against ferroptosis (Fig. 4) (Santoro, 2020).

### Ferroptosis in acute inflammation

Ferroptosis underlies multiple pathological processes, including cancer, neurodegenerative diseases, ischemia/reperfusion injury, and inflammation (Han et al., 2020). Interestingly, ferroptosis is a relevant form of cancer cell death and reduced ferroptosis may contribute to tumor development, thus indicating that it is a tumor-suppression mechanism (Gong et al., 2022).

Excessive ferroptosis has also been linked to disorders associated with mitochondrial dysfunction, such as acute inflammation (Chen et al., 2021b). Ferroptosis is, indeed, involved in the pathogenesis of acute pancreatitis as well as acute kidney, acute lung, and acute liver injury.

Oxidative stress and glutathione depletion are involved in the onset of acute pancreatitis (Moreno et al., 2014), and pancreatic-specific Gpx4 deficiency exacerbates cerulein-induced pancreatitis in mice *via* a trypsin-dependent mechanism (Dai et al., 2020; Liu et al., 2022b). Interestingly, the circadian transcription factor BMAL1 (also known as aryl hydrocarbon receptor nuclear translocator-like) regulates the expression of key ferroptotic inhibitory genes, such as SLC7A11 and GPX4, and pancreatic-specific Bmal1-deficient mice exhibited enhanced pancreatic ferroptosis in acute pancreatitis with increased mortality, tissue injury, neutrophil infiltration, and HMGB1 release (Liu et al., 2020).

Ferroptosis may, in fact, be the trigger of the inflammatory response, as shown in acute kidney injury (Martin-Sanchez et al., 2020), thereby providing a novel therapeutic approach to inflammatory disorders. For instance, ferrostatin-1 alleviated organ damage and necrosis in acute kidney injury induced by ischemia-reperfusion (Linkermann et al., 2014), folic acid (Martin-Sanchez et al., 2017), and cisplatin (Ikeda et al., 2021).

In acute kidney injury induced by lipopolysaccharide (LPS), pretreatment with MitoQ attenuated glutathione depletion and lipid peroxidation and prevented the formation of small mitochondria characteristic of ferroptosis (Liang et al., 2022). Ferrostatin-1 or the iron chelator desferoxamine completely prevented acute liver failure and mortality induced by high-dose acetaminophen (Yamada et al., 2020), or by LPS and d-galactosamine (Huang et al., 2022).

Ferroptosis and oxidative stress also contribute to the pathogenesis of acute lung injury (Liu et al., 2021; Wu et al., 2018), as shown in several acute lung injury models of infection, radiation, ischemia-reperfusion, or chemical factors (Dar et al., 2018; Dong et al., 2020; Liu et al., 2022a; Liu et al., 2020).

### Mitochondrial regulation of ferroptosis: role of mtROS

Mitochondria contribute to the regulation of ferroptosis, and, in fact, the alteration of mitochondrial morphology is used to differentiate ferroptosis from other types of cell death (Dixon et al., 2012). Three categories of morphologies have been established, based on the mitochondria morphology and their density around the nucleus, with elongated mitochondria defining class I, those with fragmented mitochondria class II, and those with fragmented mitochondria that cluster around the nucleus class III (Jelinek et al., 2018; Neitemeier et al., 2017).

Ferroptotic cells show increased mitochondrial membrane density, volume reduction, and increased membrane potential, but they do not share any of the characteristic morphologic features observed in necrosis, apoptosis, or autophagy, that is, cytoplasmic swelling, chromatin condensation, and formation of double-membrane enclosed vesicles (DeHart et al., 2018).

Erastin is a potent ferroptosis inducer that acts by directly inhibiting X_c_^−^ and also the mitochondrial voltage-dependent anion channel (VDAC). Erastin can directly bind to VDAC2 and VDAC3, causing their opening to increase mitochondrial potential and subsequent ferroptosis; whereas knockdown of VDAC2 and VDAC3 leads to erastin resistance (DeHart et al., 2018; Yagoda et al., 2007).

Although initial investigations did not focus on the role of mtROS in ferroptosis (Dixon et al., 2012; Wang et al., 2016b), more recent studies demonstrate their contribution to this type of programmed cell death. The ROS production by mitochondria can activate the AMP-activated protein kinase (AMPK)-Unc-51 like autophagy activating kinase (ULK1) axis, increasing intracellular iron levels and lipid peroxidation, and triggering vascular inflammation and ferroptosis in endothelial cells after exposure to zinc oxide nanoparticles (Qin et al., 2021).

Furthermore, ferroptosis triggered by mtROS is considered a critical mechanism contributing to sepsis-induced cardiac injury. Thus, in H9c2 myofibroblasts treated with LPS, increased cytoplasmic Fe^2+^ levels activated the expression of siderofexin in the mitochondrial membrane, which transported cytoplasmic Fe^2+^ to the mitochondria leading to enhanced production of mtROS and ferroptosis (Li et al., 2020). Accordingly, yes-associated protein 1 can block the transport of cytoplasmic Fe^2+^ into the mitochondria *via* SFXN1, reducing the generation of mtROS and, consequently, inhibiting ferroptosis in murine lung epithelial-12 cells stimulated with LPS (Zhang et al., 2022).

In addition, ferroptosis can be triggered by inactivation of mitochondrial hydroorotate dehydrogenase, which leads to extensive mitochondrial lipid peroxidation in cells with low GPX4 levels (Fig. 4) (Mao et al., 2021). Ferroptosis can be also caused by accumulation of glutamate resulting from the inhibition of system X_c_^−^, which increases mtROS production as a consequence of enhanced Krebs cycle fueling *via* glutaminolysis (Zheng and Conrad, 2020).

For instance, glutamate enhanced ROS production and lipid peroxidation in oligodendrocytes, leading to opening of the mPTP and ferroptosis in an Sirt3-dependent mechanism, a key deacetylase of the mitochondrial antioxidant system (Novgorodov et al., 2018).

The precise mechanism of membrane rupture in ferroptosis and the contribution of mtROS to ferroptosis are still unclear and require further research.

## Pyroptosis

NACHT, LRR, and PYD domains-containing protein 3 (NLRP3) is an intracellular sensor of cellular stress that detects endogenous DAMPs and exogenous pathogen-associated molecular patterns or environmental irritants, thereby assembling and activating the NLRP3 inflammasome, which triggers caspase-1-dependent cleavage and activation of pore forming gasdermin (Liu et al., 2018a; Man et al., 2017; Swanson et al., 2019).

Gasdermins, which have an N-terminal cell death domain and a C-terminal autoinhibition domain, trigger pyroptosis, a pro-inflammatory form of lytic programmed cell death (Broz et al., 2020; Swanson et al., 2019). Gasdermin is cut of its C-terminus inhibitory domain by caspase-1 (He et al., 2015; Shi et al., 2015), then binds by its N-terminal domain phosphatidylinositol phosphates and phosphatidylserine in the inner leaflet of the plasma membrane, oligomerizes, and inserts into the plasma membrane in which it forms large pores that cause membrane permeabilization and cell killing (Broz et al., 2020; Ding et al., 2016).

**FIG. 5.**
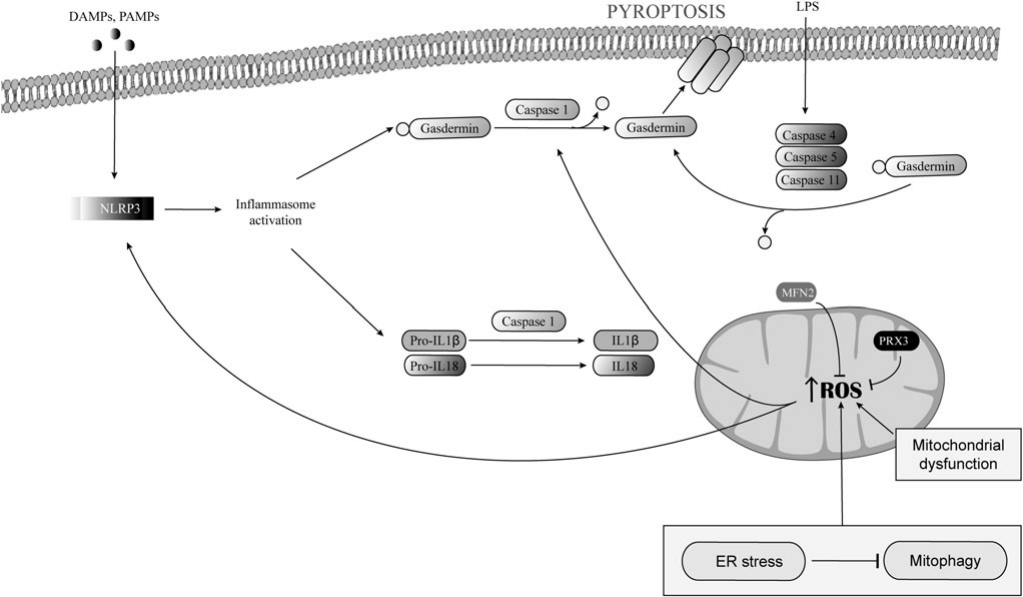
**Regulation of pyroptosis by mtROS.** DAMPs and PAMPs activate the NLRP3 inflammasome, which triggers caspase-1-dependent release of IL-1β and IL-18 and cleavages gasdermin. Cleaved gasdermins oligomerizes and inserts into the plasma membrane causing membrane permeabilization and cell killing. Pyroptosis can also be induced by an inflammasome independent pathway through caspase-11, caspase-4, and/or caspase-5. The ER stress response and mitochondrial dysfunction promotes mtROS production. The mtROS enhances NLRP3 inflammasome activation and oxidizes gasdermin D, which enhances its cleavage by caspase-1. Mitofusin 2 and PRX3 lowered mtROS and suppressed NLRP3 activation and pyroptosis. DAMPs, damage-associated molecular patterns; ER, endoplasmic reticulum; IL, interleukin; PAMPs, pathogen-associated molecular patterns.

The ROS production is one of the crucial parameters in NLRP3 activation that creates a feedback loop enhancing pyroptosis (Fig. 5) (Cruz et al., 2007; Dominic et al., 2022; Dostert et al., 2008; Li et al., 2019; Tschopp and Schroder, 2010).

Pyroptosis can also be induced by an inflammasome independent pathway that uses caspase-11 or caspase-4 in mice and caspase-5 in humans (Aglietti et al., 2016; Kayagaki et al., 2015; Kesavardhana et al., 2020; Shi et al., 2015), which are activated directly by pathogen-encoded molecules such as bacterial LPS (Hagar et al., 2013; Kesavardhana et al., 2020; Shi et al., 2015).

Similar to caspase-1, activated inflammatory caspases cleave gasdermin D, thus triggering pore formation and pyroptosis (Fig. 5) (Aglietti et al., 2016; Kayagaki et al., 2015; Liu et al., 2016; Shi et al., 2015; Wang et al., 2020). Caspase-8 may also cleave gasdermin D to induce pyroptosis in response to chemotherapeutic drugs or to some pathogens (Orning et al., 2018; Wang et al., 2017).

### Pyroptosis in acute inflammation

Pyroptosis is the inflammatory programmed cell death induced by inflammatory caspases, namely human and mouse caspase-1, human caspase-4 and caspase-5, or mouse caspase-11, which trigger secretion of inflammatory cytokines IL-1β and IL-18 during pyroptosis (Man et al., 2017; McKenzie et al., 2020; Place and Kanneganti, 2019).

IL-1β is a potent endogenous pyrogen that stimulates fever, leukocyte tissue migration, and expression of diverse cytokines and chemokines. IL-18 induces IFNγ production and is important for the activation of T cells, macrophages, and other cell types involved in immune responses (Bergsbaken et al., 2009). In addition, plasma-membrane rupture in cells undergoing pyroptosis causes the release of other proinflammatory intracellular contents that exacerbate the inflammatory response (Man et al., 2017).

Pyroptosis protects against infection and acts as a host defense mechanism in a wide range of microbial infections, including those be *Salmonella typhimurium*, *Shigella flexneri*, *Listeria*, and *Legionella pneumophila* (Pan et al., 2022). Early in infection, pyroptosis helps to eliminate infected cells and leads to activation and recruitment of immune cells and innate response (Wu et al., 2022). During persistent infection, continued caspase-1-dependent inflammation promotes the development of adaptive immune responses that lead to resolution of infection (Bergsbaken et al., 2009).

Although low grade or moderate pyroptosis facilitates homeostasis, overactivated pyroptosis can be detrimental (Wu et al., 2022). Thus, in inflamed tissues, excessive activation of pyroptosis contributes to persistent inflammation and notably impacts inflammatory disease progression (Rao et al., 2022). Indeed, pyroptosis is involved in the pathogenesis of many inflammatory diseases, such as myocardial infarction, cerebral ischemia, inflammatory bowel disease, acute lung injury, acute pancreatitis, and endotoxic shock (Bergsbaken et al., 2009; Coll et al., 2022).

### Role of mitochondria and mtROS in pyroptosis

Mitochondria are critical in the initiation and regulation of canonical NLRP pathway (Fig. 5) (Del Re et al., 2019; Liu et al., 2018a; Nakahira et al., 2011; Shimada et al., 2012; Zhong et al., 2018). Mitochondrial dysfunction and the release of mtROS, mtDNA, and cardiolipin into the cytosol are, indeed, crucial upstream events in the activation of the NLRP3 inflammasome (Elliott and Sutterwala, 2015; Swanson et al., 2019).

Many NLRP3 activators trigger a loss of mitochondrial membrane potential on mitochondrial Ca^2+^ uptake and mtROS production (Triantafilou et al., 2013; Zhong et al., 2016; Zhou et al., 2011). Activated caspase-1 contributes to mitochondrial dysfunction in pyroptosis by causing fragmentation, mtROS production, mitochondrial membrane potential dissipation and permeabilization, and mitophagy inhibition by cleavage of the mitophagy regulator Parkin (Yu et al., 2014). Inhibition of complex I and complex III by rotenone and antimycin A, respectively, induces mtROS and NLRP3 activation (Nakahira et al., 2011; Zhou et al., 2011), and imiquimod-induced NLRP3 activation is dependent on mtROS production (Groß et al., 2016).

Autophagy inhibits NLRP3 inflammasome activation through p62-dependent clearance of damaged mitochondria (Zhong et al., 2016), and inhibition of mitophagy exacerbates the activation of the NLRP3 inflammasome and of pyroptosis by lack of clearance of dysfunctional mitochondria (Yu et al., 2014; Zhou et al., 2011).

The ER stress response caused by inactivation of the ER stress regulator XBP1 promotes mtROS production by impairing mitophagy, which enhances NLRP3 inflammasome activation and pyroptosis of hepatocytes in acute liver injury (Liu et al., 2022b), whereas up-regulation of PGC-1α and mitofusin 2 lowers mtROS and suppresses NLRP3 activation and pyroptosis in alcoholic liver disease (Kai et al., 2020). By restraining mtROS production, PRX3 mitigates NLRP3-dependent pyroptosis in acetaminophen-induced liver injury (Wang et al., 2021).

Circulating mtDNA can act as a DAMP for NLRP3 activation (Nakahira et al., 2011; Shimada et al., 2012; Zhong et al., 2018). mtDNA oxidizes and is released into the cytosol on exposure to NLRP3 activators, and specifically activates the NLRP3 inflammasome (Shimada et al., 2012). mtROS oxidize gasdermin D, which enhances its cleavage by caspase-1 (Wang et al., 2019).

Other observations have linked mtROS production to the activation of pyroptosis. For instance, the Ragulator-Rag complex acts *via* mTORC1 to promote gasdermin D oligomerization and pore formation in macrophages by generating mtROS (Evavold et al., 2021), and the noncanonical activation of gasdermin D by caspase-4 triggers mtROS in macrophages upstream of the NLRP3 inflammasome (Platnich et al., 2018). Moreover, TRAF3 triggers mtROS production and pyroptosis in macrophages by promoting ULK1 ubiquitination and degradation.

Mitochondria may also act as docking sites for inflammasome assembly (Swanson et al., 2019). Under resting conditions, NLRP3 is located to the cytosolic face of the ER membrane, and when activated it relocates to the mitochondria outer membrane by binding cardiolipin, and during RNA viral infection to mitochondrial antiviral signaling protein and to mitofusin 2 (Elliott et al., 2018; Franchi et al., 2014; Ichinohe et al., 2013; Iyer et al., 2013; Park et al., 2013; Subramanian et al., 2013; Zhou et al., 2011).

## Modulation of Lytic Programmed Cell Death by Mitochondrial Antioxidants: Significance and Future Perspectives

The ability of mitochondrial targeted antioxidants, such as MitoQ and MitoTEMPO, to modulate lytic programmed cell death in the context of acute inflammation has been tested in different experimental animal models (Table 1). Nevertheless, their efficacy relies on adequate dosage and timing, and it also depends on the basal condition of the organism.

**Table 1.** Ability of Mitochondrial Targeted Antioxidants to Modulate Lytic Programmed Cell Death in the Context of Acute Inflammation in Different Experimental Animal Models

Antioxidant	Dose	Effect on programmed cell death	Experimental model	Function/mechanism	References
MitoTEMPO	20 mg/kg (i.p.)	Necroptosis prevention	Murine model of APAP hepatotoxicity	Avoid mitochondrial peroxynitrite formation	Du et al. (2019)
MitoTEMPO	25 mg/kg (i.p.)	Necroptosis prevention	Murine model of acute pancreatitis (cerulein) in sulfiredoxin KO mice	Avoid Prx3 hyperoxidation	Rius-Pérez et al. (2022)
MitoQ	10 mg/kg (2 doses)	No change	Murine model of acute pancreatitis (cerulein)	Avoid mitochondrial ROS formation	Huang et al. (2015)
MitoQ	25 mg/kg (2 doses)	Tendency to increase necrosis	Murine model of acute pancreatitis (cerulein)	Avoid mitochondrial ROS formation	Huang et al. (2015)
XJB-5-131	0.1 μ*M*	Ferroptosis prevention	HT-1080 cells	Avoid mitochondrial lipid peroxidation	Krainz et al. (2016)
MitoQ	1 μ*M*	Ferroptosis prevention	HT22 cells	Avoid mitochondrial ROS formation	Jelinek et al. (2018)
MitoQ	1 μ*M*	Ferroptosis prevention	MEF cells	Avoid mitochondrial ROS formation	Jelinek et al. (2018)
MitoTEMPO	5 mg/kg (i.p.)	Ferroptosis prevention	Murine model of DOX-induced cardiomyopathy	Avoid mitochondrial lipid peroxidation	Fang et al. (2019)
SS-31	50 or 100 μg/mL	Pyroptosis prevention	Nucleus pulposus cells	Avoid mitochondrial ROS formation	Peng et al. (2021)
MitoTEMPO	20 mg/kg (i.p.)	Pyroptosis prevention	Murine model of LPS-induced Sepsis	Avoid mitochondrial ROS formation	Wang et al. (2022)

DOX, doxorubicin; LPS, lipopolysaccharide; Prx3, peroxiredoxin 3; ROS, reactive oxygen species.

Treatment with MitoTEMPO at the dose of 20 mg/kg protected against acute liver injury by preventing necroptosis; however, it switched the mode of cell death to apoptosis during the late phase of this disease (Du et al., 2019). In acute pancreatitis, treatment with MitoTEMPO at the dose of 25 mg/kg abrogated necroptosis in mice lacking SRX, which exhibited hyperoxidation of mitochondrial PRX3 as an index of high mitochondrial H_2_O_2_ generation (Rius-Pérez et al., 2022).

In wild-type mice with acute pancreatitis, MitoQ treatment at both low (10 mg/kg) and high doses (25 mg/kg) reduced pancreatic edema and neutrophil infiltration (Huang et al., 2015). However, MitoQ was not efficient against necrosis in wild-type mice with acute pancreatitis, and unexpectedly even the higher dose tended to cause more necrosis (Huang et al., 2015).

MitoQ at 1 μ*M* abolished mtROS formation and lipid peroxidation, preserved mitochondrial function, and prevented RSL3-induced ferroptosis in neuronal HT22 cells and mouse embryonic fibroblasts despite the loss of GPX4 activity (Jelinek et al., 2018). Interestingly, MitoQ alone in the absence of RSL did not affect cell viability but triggered mitochondrial fragmentation (Jelinek et al., 2018). MitoTEMPO at the dose of 5 mg/kg also inhibited lipid peroxidation, iron-dependent ferroptosis in cardiomyocytes, and cardiomyopathy induced by doxorubicin without affecting iron levels (Fang et al., 2019).

On the other hand, the mitochondria-targeted nitroxide XJB-5-131, an ROS scavenger, protected against mitochondrial lipid peroxidation and erastin-induced ferroptosis (Krainz et al., 2016).

Regarding pyroptosis, the mitochondrial antioxidant peptide SS-31 attenuated LPS-induced pyroptosis of nucleus pulposus cells by scavenging mtROS and inhibiting NLRP3 inflammasome (Peng et al., 2021). In addition, mito-TEMPO pretreatment at a dose of 20 mg/kg markedly diminished mtROS, restored mitochondrial size and function, and reduced LPS-induced liver inflammation and injury at least in part by preventing pyroptosis (Wang et al., 2022).

Therefore, the prevention of lytic programmed cell death by mitochondrial antioxidants provides a strong proof for the involvement of mtROS in this type of cell death. However, the abolishment of mtROS in healthy cells and under basal conditions or by using high doses of mitochondrial antioxidants may affect mitochondrial dynamics and might even abrogate protective mechanisms triggered by physiological low levels of mtROS, thus enhancing cell death under certain conditions.

Hence, the therapeutic use of mitochondrial antioxidants in acute inflammation should follow the guidelines of personalized and precision medicine, and it would require to establish the appropriate dosage, timing, and restrictions due to potential detrimental side effects according to the condition of the subject.

## Concluding Remarks and Future Perspectives

The mtROS are definitively involved in the activation and execution of necroptosis, ferroptosis, and pyroptosis, all forms of lytic RCD that unquestionably contribute to inflammatory disorders. mtROS together with the necrosome and p53, the inflammasome and gasdermin D, and GPX4 deficiency operate positive feedback loops in the activation of lytic RCD pathways, and in the tissue propagation of cell death.

The precise mechanism of membrane rupture in ferroptosis and the exact contribution of mtROS to ferroptosis are still unclear and require further research. Mitochondrial antioxidants provide new promising therapeutic approaches for acute inflammatory disorders. To allow their use, to optimize their therapeutic potential, and to avoid adverse side by compromising beneficial mtROS signaling, it will be critical to understand when mtROS cause detrimental effects and when and how they promote survival and beneficial signaling.

## Abbreviations Used

ACD: accidental cell death
BHA: butylated hydroxyanisole
BMAL1: brain and muscle ARNT-like 1
cfDNA: cell free DNA
CHMP4B: charged multivesicular body protein 4B
cIAP: cellular inhibitor of apoptosis protein
CoQ10: ubiquinone
CoQH10: ubiquinol
CXCL12: chemokine (C-X-C motif) ligand 12
CYLD: CYLD lysine 63 deubiquitinase
CypD: cyclophilin D
DAMPs: damage-associated molecular patterns
DHODH: hydroorotate dehydrogenase
DOX: doxorubicin
DRP1: dynamin-related protein 1
ER: endoplasmic reticulum
ESCRT: endosomal sorting complex required for transport
FADD: TRADD-FAS-associated death domain protein
Fas: Fas cell surface death receptor
FasL: Fas Ligand
FSP1: NAD(P)H/ferroptosis suppressor protein-1
GPX4: glutathione peroxidase 4
HMGB1: high mobility group box 1
HSP90: heat shock protein 90
IFNRs: interferon receptors
IL: interleukin
LOX: iron-containing lipoxygenase
LPS: lipopolysaccharide
*Lrrk2*: leucine-rich repeat kinase 2
MLKL: pseudokinase mixed-lineage kinase domain–like
mPTP: mitochondrial permeability transition pores
mtDNA: mitochondrial DNA
mTORC1: mammalian target of rapamycin complex 1
mtROS: mitochondrial reactive oxygen species
NDUFB8: NADH dehydrogenase (ubiquinone) 1 beta subcomplex 8
NF-κB: nuclear factor kappa-light-chain-enhancer of activated B cells
NLRP3: NACHT, LRR, and PYD domains-containing protein 3
NOX: NADPH oxidases
PAMPs: pathogen-associated molecular patterns
PDC: pyruvate dehydrogenase complex
PGAM5: phosphoglycerate mutase 5
PGC-1α: peroxisome proliferator-activated receptor gamma coactivator 1-alpha
PITPα: phosphatidylinositol transfer protein alpha
PRX: peroxiredoxin
RAC1: Ras-related C3 botulinum toxin substrate 1
RCD: regulated cell death
RET: reverse electron transport
RIPK1: receptor-interacting protein kinase 1
RIPK3: receptor-interacting protein kinase 3
ROS: reactive oxygen species
RSL3: RAS-selective lethal 3
SLC3A2: solute carrier family 3 member 2
SLC7A11: solute carrier family 7 member 11
SRX: sulfiredoxin
TEMPOL: 4-hydroxy-2,2,6,6-tetramethylpiperidine-1-oxyl
TNFα: tumor necrosis factor α
TNFR1: tumor necrosis factor alpha receptor 1
TRADD: TNFR1-associated death domain
TRAF: tumor necrosis factor receptor–associated factor
TRAIL: TNF-related apoptosis-inducing ligand
ULK1: Unc-51 like autophagy activating kinase
VDAC: voltage-dependent anion channel
XBP1: X-box binding protein 1
